# Efficacy, safety, and perioperative outcomes of holmium laser enucleation of the prostate—a comparison of patients with lower urinary tract symptoms and urinary retention

**DOI:** 10.1007/s10103-020-03170-4

**Published:** 2020-10-30

**Authors:** Pawel Trotsenko, Christian Wetterauer, Philipp Grimsehl, Tilmann Möltgen, Susan Meierhans, Lukas Manka, Helge Seifert, Stephen Wyler, Maciej Kwiatkowski

**Affiliations:** 1 Department of Urology, Cantonal Hospital Aarau, Aarau, Switzerland; 2 Department of Urology, University Hospital Basel, Spitalstr. 21, 4031 Basel, Switzerland; 3 Department of Urology, Academic Hospital Braunschweig, Braunschweig, Germany

**Keywords:** Holmium laser enucleation of the prostate, HoLEP, Lower urinary tract symptoms, LUTS, Urinary retention

## Abstract

Holmium laser enucleation of the prostate (HoLEP) is a valid treatment option to relieve bladder outlet obstruction in patients with large prostate volumes (PV). Its efficacy, tolerability, and safety are comparable to the ones of other laser treatments of the prostate and resection techniques. However, safety and efficacy of HoLEP have not been compared between patients with and without preoperative urinary retention. We included 350 patients (mean age 71.2 years) who had undergone HoLEP due to lower urinary tract symptoms (LUTS) or urinary retention caused by prostatic hyperplasia. We evaluated the differences in peri- and postoperative outcomes and complications between patients with and patients without preoperative urinary retention. The mean PV was 115 cm^3^. PV was > 100 cm^3^ in 61.9% and < 100 cm^3^ in 38.1% of the patients. Perioperative complications occurred in 23 patients (6.6%), 15 of which (4.3%) required operative revision. We found no significant differences in terms of complication rates between patients with PV > 100 cm^3^ and patients with PV < 100 cm^3^. Mean catheterization-duration was 3.3 days. Preoperatively, 140 patients (40%) had a suprapubic or transurethral indwelling catheter; they did not differ from patients without preoperative catheter regarding postoperative catheter removal success rate, early postoperative complications, and functional outcomes. Prostate cancer was diagnosed in 43 patients (12.3%). Median postoperative PSA-decline was 6.1 ug/l (89.8% drop). HoLEP is a safe and effective treatment for patients with LUTS or urinary retention and large PV. PV > 100 cm^3^ was not associated with higher complication rates or successful catheter-removal. Furthermore, functional outcomes were independent of preoperative catheterization.

## Introduction

Affecting nearly 75% of men in the seventh decade, benign prostatic hyperplasia (BPH) is one of the most common urological diseases that is associated with major impact on the quality of life (QoL) of patients as well as with substantial costs [1, 2]. Although conservative therapy is initially successful in many patients suffering from lower urinary tract symptoms (LUTS), surgical therapy becomes necessary when medical therapy fails.

Dependent on the prostate volume (PV), different surgical techniques are used [2]. For volumes of less than 80–100 cm^3^, transurethral resection of the prostate (TUR-P) is still considered the gold standard [2–4]. In patients with larger PV, open prostatectomy (OP) is the most common approach used in many parts of Europe [5].

In the course of the introduction of modern laser technologies, holmium laser enucleation of the prostate (HoLEP) has emerged as a minimally invasive alternative treatment option for PV exceeding 80 cm^3^. Several randomized controlled trials could demonstrate that HoLEP and OP are equally effective [5, 6]. Among the available transurethral procedures for benign prostatic hyperplasia (BPH), HoLEP was associated with the lowest cumulative perioperative complication rate [7], and was shown to be superior as compared to OP in terms of blood loss and catheterization times [5, 6]. Following this, HoLEP has been included in the European LUTS guidelines [2, 8–12].

However, there is limited data on the effectiveness of HoLEP in patients with preoperative urinary retention and catheterization [3].

Therefore, the primary aim of this study was to evaluate the efficacy of HoLEP in terms of early postoperative outcome in patients with LUTS and urinary retention. Furthermore, we assessed the safety concerning perioperative complications, as well as the effect of PV on perioperative outcome.

## Materials and methods

### Patients

In this retrospective study, we reviewed the medical charts of 350 consecutive patients who had presented with symptomatic BPH and had undergone HoLEP surgery at our institution between June 2012 and May 2019. On average, about 50–70 procedures are performed per year at our institution.

Patients with neurogenic bladder or with previously diagnosed significant prostate cancer (PCa) were excluded from the analysis. If clinically indicated, preoperative magnetic resonance imaging and a consecutive biopsy of the prostate were performed. Follow-up was completed 2 months after surgery.

We assessed pre-, peri-, and postoperative parameters like PV, maximal flow rate (Q_max_), postvoid-residual urine volume (PVR), voiding volume (VV) and PSA, duration of catheterization, catheterization due to urinary retention, and complications and incidence of PCa. Approval by the local Ethics Committee was granted (Ethikkommission Nordwest- und Zentralschweiz; ID 2019-01275), and the study was performed in accordance with the Declaration of Helsinki. The Ethics Committee has waived the need to obtain written informed consent

All study results were reported according to the “Strengthening the Reporting of Observational Studies in Epidemiology” (STROBE) guideline [13].

### Equipment and surgical procedure

The equipment for HoLEP comprised a 100-W holmium laser (VersaPulse, Lumenis©), a 550-μm end-firing fiber (Slimline 550, Lumenis©), a modified continuous-flow resectoscope (25F), a tissue morcellator (Piranha, Richard Wolf©), continuous saline irrigation, and a video system. Power settings were adjusted to 1.9 J at 53 Hz.

All procedures were performed by experienced surgeons (TM, PG), or under close supervision (SM), using a 2- or 3-lobe technique under general anesthesia. Technical details have been described previously [14, 15]. Following enucleation, the prostate-tissue was recovered from the bladder using a morcellator and obtained for histological analysis. Coagulation was achieved by defocusing the laser fiber. We performed bipolar coagulation of the entire prostatic fossa and established continuous bladder irrigation. According to our standard protocol, bladder irrigation was gradually reduced on postoperative (po) day 1; catheter-removal took place on po day 2, with a subsequent assessment of Q_max_ and PVR. Patients were discharged on po day 3 without specific medication. Follow-up was performed 2 months after surgery including PSA, PVR and uroflowmetry.

### Statistical methods

Statistical analyses were performed with SPSS Statistics 24.0 (IBM©). We used a Student *t* test for the analysis of our datasets, and a two-sided *t* test for the comparison of two groups. All tests were performed at a significance level of *α* = 0.05. Risk stratification was described by odds ratio. PSA decline was presented by linear regression or Pearson correlation coefficient. All data are presented as mean, ± standard deviation of the mean (SD), and median. Follow-up data were assessed only for patients with available pre- and postoperative values.

## Results

**Fig. 1:**
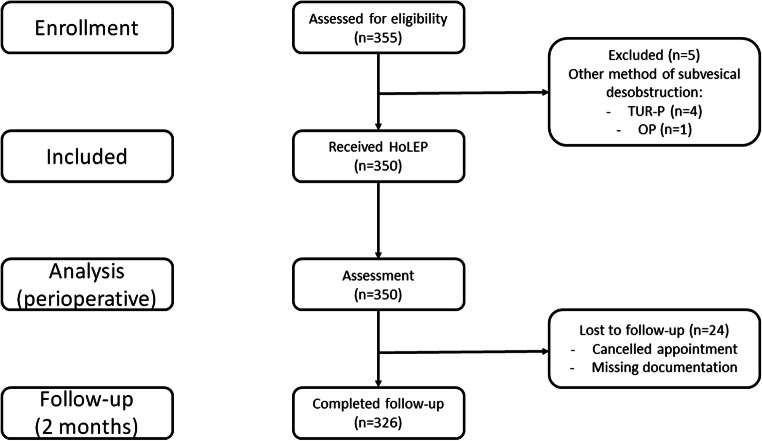
Flowchart of enrollment, inclusion, and follow-up

Between June 2012 and May 2019, HoLEP was successfully performed in a total of 350 patients. A flowchart of the study course (recruitment, enrollment, and follow-up) is presented in Fig. 1. Due to a lack of documentation or cancelled appointments, 24 of the 350 patients (6.9%) were lost to follow-up 2 months after surgery. Patients’ baseline and perioperative characteristics are summarized in Table 1.

**Table 1:** Baseline characteristics and perioperative data

Patients (*n* = 350)	
Parameter	Mean ± SD (range)
Age (years)	71.2 ± 8 (48.3–90.5)
Transvesical PV (cm^3^)	115.1 ± 41.8 (30–320)
Total PSA (μg/l)	9.2 ± 9.9 (0.3–88)
Q_max_ (ml/sec)	9.4 ± 4.7 (0–22)
PVR (ml)	196.7 ± 221.3 (0–1500)
Voiding volume (ml)	166.9 ± 114.1 (0–640)
Operation time (min)	82.1 ± 35.6 (11–251)
Tissue retrieved (g)	69.2 ± 35 (9–279)
Resection velocity (g/min)	0.9 ± 0.4 (0.1–4)
Catheterization time (days)	3.3 ± 5.2 (1–61)

*PV*, prostate volume; *PSA*, prostate-specific antigen; *Q*_*max*_, maximum flow rate; *PVR*, post-void residual

Baseline and outcome measures of all functional parameters and the PSA course are demonstrated in Table 2. Postoperatively, we could detect significant improvements of Q_max_, PVR, and VV, as well as a significant PSA-drop (*p* < 0.05).

**Table 2:** Functional parameters and PSA pre- and postoperatively

Parameter	No. of patients	Baseline	2 months	*p* value
Q_max_ (ml/sec)	115	10.2 ± 4.6 (1.9–22)	28.6 ± 15.2 (6.8–80)	< 0.001
PVR (ml)	217	198.4 ± 220.5 (0–1500)	17.2 ± 37.3 (0–340)	< 0.001
Voiding volume (ml)	138	175.9 ± 120.3 (0–640)	245.7 ± 179.8 (13–1124)	< 0.001
Total PSA (μg/l)	209	9.4 ± 9.5 (0.3–78.8)	1.6 ± 4.1 (0.1–39)	< 0.001

All patients with documented baseline and follow-up parameters included. Data shown as mean ± standard deviation (range). *Q*_*max*_, maximum flow rate; *PVR*, post-void residual; *PSA*, prostate-specific antigen

Prior to surgery, 140 of the 350 patients (40%) had a suprapubic or transurethral indwelling catheter. The comparison of patients with and patients without prior catheterization revealed no significant differences in terms of postoperative catheter-removal success rate or functional parameters. The only significant difference found was a longer postoperative catheterization time in the urinary retention group. A detailed comparison of all baseline and outcome measures for patients with and patients without prior catheterization is displayed in Table 3.

**Table 3:** Baseline characteristics and outcome with/without preoperative catheterization

Parameter	No catheter	Catheter	*p* value
Patients *n* (%)	210 (60)	140 (40)	-
Complications *n* (%)	13 (6.2)	10 (7.1)	0.74
Blood transfusion *n* (%)	5 (2.4)	3 (2.1)	0.88
TUR—evacuation/coagulation	8 (3.8)	7 (5)	0.6
Dismissal with catheter *n* (%)	6 (2.9)	10 (7.1)	0.76
Age (years)	70.2 ± 7.4 (49.1–86.2)	72.6 ± 8.6 (48.3–90.5)	0.03
Transvesical PV (cm3)	111.9 ± 43.6 (30–320)	119.9 ± 38.5 (35–220)	0.048
Catheterization time (days)	2.8 ± 4.4 (1–61)	4 ± 6.1 (1–41)	0.03
Q_max_ (ml/sec)—baseline	10.3 ± 4.4 (0–22)	5.2 ± 3.6 (0–16.3)	< 0.001
Q_max_ (ml/sec)—follow-up	26 ± 14.2 (4.4–80)	25.4 ± 13.9 (4.6–71)	0.24
PVR (ml)—baseline	143.7 ± 124.3 (0–600)	399 ± 356 (20–1500)	< 0.001
PVR (ml)—follow-up	16.4 ± 33.4 (0–340)	22.3 ± 57.5 (0–464)	0.23
VV (ml)—baseline	179.8 ± 118.3 (150–640)	107.8 ± 66.9 (0–247)	0.02
VV (ml)—follow-up	221.5 ± 184 (13–800)	233.4 ± 192.5 (13–1124)	0.36

Data shown as mean ± standard deviation (range)

*PV*, prostate volume; *Q*_*max*_, maximum flow rate; *PVR*, post-void residual; *VV*, voiding volume

Overall, 23 of the 350 patients (6.6%) developed a total of 27 early postoperative complications. Blood transfusion was necessary in 8/350 patients (2.3%), 4/350 (1.1%) developed postoperative sepsis, and 15/350 (4.3%) required early operative revision (evacuation of vesical tamponade and/or coagulation). According to the Clavien-Dindo classification, 12 complications were categorized as grade II and 15 as grade IIIb. Complications occurred in 20 of the 217 patients (9.22%; mean PV 136.9 cm^3^) with PV > 100 cm^3^ and in 7 of the 133 patients (5.26%; mean PV 79.7 cm^3^) with PV < 100 cm^3^. The comparison of these two groups revealed no significant difference regarding the occurrence of complications (OR 0.57; 95% CI 0.24–1.39; *p* = 0.22. In 6 of the 217 patients (2.8%) with PV > 100 cm^3^, a blood transfusion was indicated. Preoperative catheterization had no impact on the perioperative complication-rate (OR 0.87; 95% CI 0.37 to 2.03; *p* = 0.74). The median decline of PSA after HoLEP was 6.1 ng/ml (89.8%). The amount of removed tissue significantly correlated with the degree of PSA decline (Pearson’s *r* = 0.3). Preoperatively, 138 of the overall 350 patients (39.4%) received prostate biopsy due to elevated PSA values and/or a suspicious digital rectal examination (DRE). A clinically insignificant prostate carcinoma was detected in 24 of these 138 patients (17.4%), and the biopsy revealed no malignancy in 114 of these 138 patients (82.6%).

Histological work-up of the resected tissue revealed prostate cancer in 43 of the overall 350 patients (12.3%), 12 of which (27.9%) were clinically significant (Gleason score > 6). Twenty-three of these 43 patients (53.5%) had received no biopsy prior to surgery, 11 (25.6%) were diagnosed with benign histology upon prior biopsy, and 9 (20.9%) had been diagnosed with insignificant carcinoma by preoperative biopsy. In 15 of those 24 patients (62.5%) with pre-diagnosed insignificant carcinoma, histological work-up of resected tissue revealed no signs of malignancy.

## Discussion

This study demonstrates that HoLEP is a safe and reliable procedure that provides excellent postoperative outcome in both patients with LUTS and patients with urinary retention. Baseline characteristics of our cohort in regard to age, PSA, and PV are representative and comparable with other studies [5, 16–19].

Several long-term studies have already demonstrated the safety and efficacy of this procedure and have shown that it is comparable with TUR-P or OP [7, 17, 18, 20–22]. In line with these results, we could demonstrate significant postoperative improvements in objective voiding measures like Q_max_, PVR, and VV.

Our duration of catheterization (3.3 days) was slightly longer compared to the times measured in other HoLEP cohorts [3, 6, 10, 20, 23], which is most likely due to the standardized postoperative protocol at our institution with one day of bladder irrigation; however, the catheterization times are still shorter than those measured after OP, with a reported and a mean catheterization time of 6.1 days [5, 6, 9, 19, 24]. Our mean operation time, including morcellation time, of 82.5 min is shorter, and the amount of retrieved tissue (mean 69.2 g) is smaller than the ones reported in other HoLEP cohorts [3, 5, 19, 20, 24]. Notably, our series used the weight “only” without compensating for tissue estimated to be lost to vaporization [24]. Nevertheless, we achieved similar functional outcomes and efficiency in terms of resection velocity [5, 23, 25, 26].

So far, only one study reported on the influence of preoperative catheterization on outcomes after HoLEP [3]. Recently, Tang et al. [27] have analyzed efficacy and outcome of HoLEP in patients with urinary retention due to advanced PCa, and other studies have not performed direct comparisons [23, 28].

This study provides evidence for the excellent functional outcomes after HoLEP, independent of catheterization prior to surgery [3]. Our patients with and without prior urinary retention displayed significant differences in baseline parameters. Yet, the comparison of postoperative functional parameters after HoLEP, like Q_max_, VV, or PVR, revealed no significant difference between the two groups. These findings highlight the efficacy of HoLEP in patients with urinary retention, and corroborate previous findings [3]. Early operative revision for gross hematuria and/or bladder tamponade was required in 15 of our 350 patients (4.3%), a rate that is in line with rates of up 5% reported by Kuntz et al. [16], however higher than the 1.4% reported by Ahyai et al. [7].

According to current literature [7], postoperative sepsis overall occurs in a range of 0–3.0% after transurethral treatment and our reported rate of 1.1% for the development of postoperative sepsis highlights the favorable safety profile of HoLEP. Additionally, we assessed whether the presence of a PV > 100 cm^3^ had an influence on the rate of associated complications; we detected no increased risk for early postoperative complications. This finding is in line with various studies supporting the safety of HoLEP, independently of PV [6, 20]. Only Zell et al. [29] have reported elevated complication rates after HoLEP for PV exceeding 200 cm^3^.

The amount of tissue removed by HoLEP led to a PSA decline of 6.1 ng/ml (89.8%) in our series. The association between tissue loss and PSA drop of 60–90% after HoLEP has been reported earlier [20], and underlines the efficacy of HoLEP [22].

Despite no clinical suspicion of cancer and/or negative prostate-biopsy prior to the intervention, PCa was detected in 43 of our 350 patients (12.3%), 12 of which (3.4%) were clinically relevant. In line with this result, Krambeck et al. have reported that 106 of their overall 1000 cases (10.1%) were diagnosed with PCa after the intervention [20]. These results stress the importance of a preoperative PCa screening, if considered clinically relevant for the individual patient.

This study is limited due to its retrospective nature, the single-center setting, and short follow-up of only 2 months. Moreover, pre- and postoperative functional data as well as PSA values were not available for all patients. Therefore, our findings should be validated in larger cohorts and preferably in a prospective setting.

To the best of our knowledge, this is one of the largest reported HoLEP cohorts in Europe [5, 16–19], and the first HoLEP series from Switzerland.

Furthermore, this is the largest study that compared HoLEP outcomes in patients with and without urinary retention; as such, we provide further evidence, on top of the only available prior report [3], for the efficiency and safety of HoLEP, regardless of preoperative retention status. In summary, our series affirms the safety, efficiency, and efficacy of HoLEP as treatment alternative for LUTS and urinary retention.

## Conclusions

In conclusion, HoLEP represents a safe and effective treatment alternative for patients with LUTS or urinary retention, and provides excellent functional outcome independent of the prostate volume. Importantly, a larger PV is not associated with a higher complication rate.

## Data Availability

All data are available from the corresponding author upon reasonable request.
